# Comparison of Conventional Logistic Regression and Machine Learning Methods for Predicting Delayed Cerebral Ischemia After Aneurysmal Subarachnoid Hemorrhage: A Multicentric Observational Cohort Study

**DOI:** 10.3389/fnagi.2022.857521

**Published:** 2022-06-17

**Authors:** Ping Hu, Yuntao Li, Yangfan Liu, Geng Guo, Xu Gao, Zhongzhou Su, Long Wang, Gang Deng, Shuang Yang, Yangzhi Qi, Yang Xu, Liguo Ye, Qian Sun, Xiaohu Nie, Yanqi Sun, Mingchang Li, Hongbo Zhang, Qianxue Chen

**Affiliations:** ^1^Department of Neurosurgery, Renmin Hospital of Wuhan University, Wuhan, China; ^2^Department of Neurosurgery, Affiliated Hospital of Panzhihua University, Panzhihua, China; ^3^Department of Neurosurgery, First Hospital of Shanxi Medical University, Taiyuan, China; ^4^Department of Neurosurgery, General Hospital of Northern Theater Command, Shenyang, China; ^5^Department of Neurosurgery, Huzhou Central Hospital, Huzhou, China; ^6^School of Physics and Technology, Wuhan University, Wuhan, China; ^7^School of Electronic Information and Automation, Guilin University of Aerospace Technology, Guilin, China; ^8^Department of Neurosurgery, The Second Affiliated Hospital of Nanchang University, Nanchang, China

**Keywords:** logistic regression, prediction model, delayed cerebral ischemia, subarachnoid hemorrhage, inflammatory response, machine learning

## Abstract

**Background:**

Timely and accurate prediction of delayed cerebral ischemia is critical for improving the prognosis of patients with aneurysmal subarachnoid hemorrhage. Machine learning (ML) algorithms are increasingly regarded as having a higher prediction power than conventional logistic regression (LR). This study aims to construct LR and ML models and compare their prediction power on delayed cerebral ischemia (DCI) after aneurysmal subarachnoid hemorrhage (aSAH).

**Methods:**

This was a multicenter, retrospective, observational cohort study that enrolled patients with aneurysmal subarachnoid hemorrhage from five hospitals in China. A total of 404 aSAH patients were prospectively enrolled. We randomly divided the patients into training (*N* = 303) and validation cohorts (*N* = 101) according to a ratio of 75–25%. One LR and six popular ML algorithms were used to construct models. The area under the receiver operating characteristic curve (AUC), accuracy, balanced accuracy, confusion matrix, sensitivity, specificity, calibration curve, and Hosmer–Lemeshow test were used to assess and compare the model performance. Finally, we calculated each feature of importance.

**Results:**

A total of 112 (27.7%) patients developed DCI. Our results showed that conventional LR with an AUC value of 0.824 (95%CI: 0.73–0.91) in the validation cohort outperformed k-nearest neighbor, decision tree, support vector machine, and extreme gradient boosting model with the AUCs of 0.792 (95%CI: 0.68–0.9, *P* = 0.46), 0.675 (95%CI: 0.56–0.79, *P* < 0.01), 0.677 (95%CI: 0.57–0.77, *P* < 0.01), and 0.78 (95%CI: 0.68–0.87, *P* = 0.50). However, random forest (RF) and artificial neural network model with the same AUC (0.858, 95%CI: 0.78–0.93, *P* = 0.26) were better than the LR. The accuracy and the balanced accuracy of the RF were 20.8% and 11% higher than the latter, and the RF also showed good calibration in the validation cohort (Hosmer-Lemeshow: *P* = 0.203). We found that the CT value of subarachnoid hemorrhage, WBC count, neutrophil count, CT value of cerebral edema, and monocyte count were the five most important features for DCI prediction in the RF model. We then developed an online prediction tool (https://dynamic-nomogram.shinyapps.io/DynNomapp-DCI/) based on important features to calculate DCI risk precisely.

**Conclusions:**

In this multicenter study, we found that several ML methods, particularly RF, outperformed conventional LR. Furthermore, an online prediction tool based on the RF model was developed to identify patients at high risk for DCI after SAH and facilitate timely interventions.

**Clinical Trial Registration:**

http://www.chictr.org.cn, Unique identifier: ChiCTR2100044448.

## Introduction

Aneurysmal subarachnoid hemorrhage (aSAH) is a severe acute cerebrovascular disorder resulting in high morbidity and mortality; roughly 50% of aSAH survivors have permanent neurological deficits (Molyneux et al., 2005; Fugate and Rabinstein, 2012). Delayed cerebral ischemia (DCI) is the most frequent complication after aSAH, affecting ~ 30% of patients, often causing serious damage because of its late diagnosis (Macdonald, 2014; Francoeur and Mayer, 2016). Hence, timely and accurate prediction of DCI is critical for the treatment and prognosis of patients with aSAH. A precise, reliable model for early prediction of DCI development is urgently needed.

Traditional logistic regression (LR) is the primary method to construct models for predicting disease outcomes. However, when LR is used for complex multivariate non-linear relationships, complex transformations are often required owing to low robustness and multicollinearity between variables (Tu, 1996). Machine learning (ML) is valuable for analyzing clinical data because it can fully employ input features and predict outcomes more accurately (Jordan and Mitchell, 2015). Several studies suggested that in DCI, ML models utilizing admission clinical characteristics have better predictive power than LR (Ramos et al., 2019; de Jong et al., 2021; Savarraj et al., 2021). However, the model performance is not generally high due to the incomplete clinical features. Admission clinical characteristics include baseline information, laboratory test results, and imaging data, and the fragmented application of these data may reduce predictive performance; therefore, these features must be systematically utilized. To the best of our knowledge, there is no study that utilizes relatively complete clinical features to construct ML and LR models, some of which were not compared in previous studies.

We determined several types of the currently most popular ML algorithms to achieve the following aims. First, we constructed and validated a conventional LR and several ML models based on relatively complete clinical features on admission. Second, we compared the predictive performances of the LR and ML models. Third, we established an online prediction tool based on the important features identified by the optimal model, which is convenient for clinicians and can precisely calculate the risk of DCI after aSAH.

## Methods

### Study Design and Patient Enrollment

**Figure 1:**
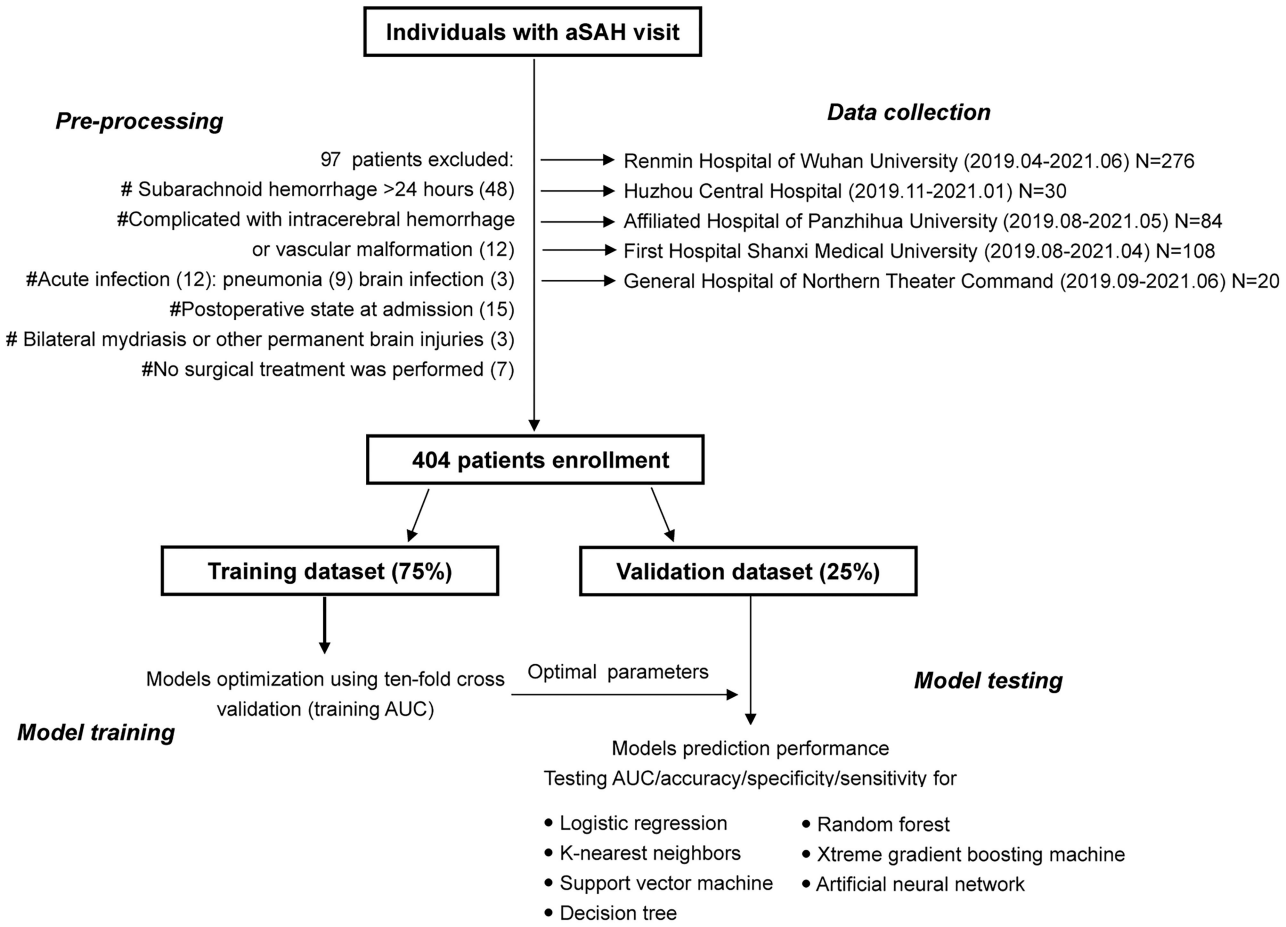
The flowchart of study design and detailed patient enrollment.

This multicenter, retrospective, observational cohort study utilized clinical data from the electronic health record system. The study participants consisted of all adult patients with aSAH within 24 h of onset who were treated in the Department of Neurosurgery from April 2019 to June 2021, Renmin Hospital of Wuhan University, Huzhou Central Hospital, Affiliated Hospital of Panzhihua University, General Hospital of Northern Theater Command, First Hospital of Shanxi Medical University. The study eventually enrolled 404 patients (Figure 1). According to SAH guidelines, aSAH was diagnosed using head computed tomography (CT), CT angiography, or digital subtraction angiography.

The inclusion criteria were: (1) spontaneous aSAH, (2) admission within 24 h after onset, (3) blood laboratory tests and head CT scans within 24 h after admission, (4) microsurgical clipping or coil embolization within 72 h after onset, and (5) DCI, which occurs within 4–30 days after aSAH.

The exclusion criteria were: (1) admission time exceeded 24 h after onset, (2) intracerebral hemorrhage or vascular malformation, (3) acute infection, (4) postoperative state on admission, (5) bilateral mydriasis or other permanent brain injuries on admission, (6) non-surgical treatment, and (7) patients who died within 3 days after admission.

### Clinical Data Collection

Patient demographic data (sex, age), medical history (hypertension, diabetes mellitus, coronary heart disease, smoking, alcohol consumption, anticoagulant treatment, and previous diseases), and clinical state on admission [World Federation of Neurosurgical Societies (WFNS), Hunt and Hess grade (HH), and modified Fisher scale (mFS)] were collected. Aneurysmal details were also recorded, including aneurysm number, location, length, neck size, and treatment. Surgical methods and laboratory tests on admission (glucose, D-dimer, as well as white blood cell [WBC], neutrophil, lymphocyte, and monocyte counts) were also utilized in this study.

### CT Value Assessment

The CT values of subarachnoid clots and cerebral edema were manually measured and collected, and measurement methods and references are provided in the Supplementary Data 1.

All CT scans were completed using a GE scanner (64-section Optimal CT680) without contrast enhancement. The following parameters were used: tube voltage, 120 kVp; tube current modulation, 300 mA; detector configuration, 64 × 0.625 mm; rotation time, 0.5 s; slice thickness, 5 mm; and collimation, 10 mm.

Regions of interest (ROIs) were manually drawn on the central area of the blood clots in representative slices by two neurosurgeons who were blinded to the patients' clinical information. The mean blood clot density in the subarachnoid space was measured in each ROI (a circle 3–8 mm across), returning the mean Hounsfield Unit (HU) value. Subarachnoid cisterns/fissures, including the lateral Sylvian fissure, anterior interhemispheric fissure, medial Sylvian fissure, suprasellar cistern, ambient cistern, and quadrigeminal cistern, were used to determine the mean HU (Woo et al., 2017; Kanazawa et al., 2020).

Regions of interest (circles 5–10 mm across) of the cerebral edema were bilaterally and symmetrically drawn on a representative CT slice. If blood clots were below the insular cortex, the ROI was drawn on the thalamus and basal ganglia. Otherwise, the ROI was drawn on the bilateral centrum semiovale (Claassen et al., 2002; Ahn et al., 2018).

### Outcome Definitions

The definition of DCI should meet at least one of the following criteria: (1) no other etiology could have caused a permanent or temporary focal neurological impairment (such as aphasia, apraxia, hemianopia, or neglect) between 4 and 14 days after aSAH; (2) the Glasgow Coma Scale score decreased by at least two points [either on one of its components (eye opening, verbal response, motor response), or on total score]; and (3) head CT scans revealed a low-density area that was not noticeable on admission or immediately after the operation, and there were no other causes except vasospasms between 4 and 30 days after aSAH (Vergouwen et al., 2010).

### Sample Size

We used the events per variable criterion with a value of 10 (Peduzzi et al., 1996) to estimate the effective sample size in this study. Our preliminary analysis indicated that nine variables were entered into a multivariable LR model. Hence, at least 90 patients with DCI should be included in the training cohort. Moreover, according to the risk of DCI occurrence after SAH, ~30% worldwide, there should be at least 300 patients in the model training cohort.

### Processing of Missing Data

This dataset included 17 patients with missing values, which accounted for <5% of the study population, so we directly used the missing value deletion method to process the data (Eekhout et al., 2012).

### Model Development

A total of 404 patients with aSAH from five medical centers were prospectively enrolled. We randomly divided the patients as training cohort (*N* = 303) and validation cohort (*N* = 101) according to a ratio of 75–25%. The training cohort was utilized to develop a conventional LR, k-nearest neighbor (KNN), support vector machine, decision tree, random forest (RF), extreme gradient boosting, and artificial neural network (ANN) models.

### Machine Learning Models Development

#### LR

The model was trained by fitting the predictor variables with *P* < 0.1 in univariate analysis to multivariate logistic analysis. We used the backward stepwise regression method based on the Akaike information criterion to select the optimal variables and constructed a final LR model. “MASS” package in R software was performed to fit the model.

#### LASSO

LASSO regression, which is suitable for analyzing high-dimensional data, was used to select the most informative prediction variables. We used the “glmnet, corrplot, caret” packages and 10-fold cross-validation to obtain the optimal λ and factors.

#### KNN

KNN model uses local geographic information in the predictive environment to predict the results of the new samples. For example, a KNN model with ten neighbors uses the ten closest observations in multidimensional space to predict the results of a new sample based on a distance assessment. The optimal K value was determined by 10-fold cross-validation and the “e1071, class, kknn, kernlab, caret” packages.

#### SVM

The uniqueness of SVM algorithms is that they mainly use data points from each result class that is closest to the class boundary or misclassified when determining the boundary structure. The radial basis function was applied in this work, and the optimal gamma value and minimum error of the SVM model were determined by 10-fold cross-validation.

#### DT

DT algorithms partition the sample data by splitting prediction features at discrete cut-points and are usually presented in the form of a tree. In this study, the decision tree algorithm uses the Gini index to determine each split's optimal variable and location. The cost complexity parameter that penalizes more complex trees is used to control the size of the final tree. Ten-fold cross-validation and “rpart, partykit, caret” packages were used to determine the minimum error value.

#### RF

RF builds a predictive model by sampling objects and variables, generating multiple decision trees, and classifying objects in turn. Finally, the classification results of each decision tree are summarized, and the mode category in all prediction categories is the category of the object predicted by the RF model. The optimal number of trees was determined using 10-fold cross-validation and “randomForest” package.

#### XGBoost

XGBoost is an optimized distributed gradient enhancement library designed to be efficient, flexible, and portable. It implements ML algorithms under the Gradient Boosting framework. The optimal parameters were determined by “xgboost” package and 10-fold cross-validation.

#### ANN

ANN is an algorithmic mathematical model that imitates the behavioral characteristics of animal neural networks and performs distributed and parallel information processing. This kind of network relies on the system's complexity, adjusts the interconnection between a mass of internal nodes to achieve the purpose of processing information, and has the ability of self-learning and self-adaptation. Ten-fold cross-validation and “caret, MASS, neuralnet, vcd” packages were conducted to determine the optimal parameters of this model.

### Dynamic Nomogram

A web-based dynamic nomogram application was then developed based on the optimal prediction variables based on optimal features. Calibration curve with 1,000 resample bootstrap was used to assess the calibration ability, and the clinical effectiveness was evaluated by decision curve analysis (DCA) and clinical impact curve (CIC). The packages “rmda, MASS, survival, ggplot2, ggridges, DynNom, and riskRegression” and “shinyapps.io” were performed to achieve this process.

### Model Performance Evaluation

We used the area under the receiver operating characteristic curve (AUC) with 95% confidence intervals (95% CIs), accuracy, balanced accuracy, confusion matrix, sensitivity, and specificity indicators in both training and validation cohorts to evaluate model performance. The AUC value was used to assess model discrimination, while the calibration curve with 10-fold cross-validation (1,000 resample) and Hosmer–Lemeshow test can reflect the model calibration performance.

### Statistical Analysis

We applied the Kolmogorov–Smirnov test to determine the data distribution before formally analyzing the data. Continuous variables analyzed using the independent *t*-test or Mann-Whitney *U*-test are presented as mean ± SD or median with interquartile range. Categorical variables analyzed using the chi-square or Fisher's exact tests are expressed as numbers (percentages). The statistical difference between the AUCs of these models was completed by DeLong test. The feature importance was calculated by Gini index using RF algorithm. The total score of all feature importance was added up to 100. A higher importance coefficient commonly indicated a stronger influence on the occurrence of DCI. For continuous variables that were important for DCI indicator, we used the Youden index to calculate the cut-off value to distinguish patients who were prone to be DCI. All statistical tests were two-tailed and *p* < 0.05 were considered statistically significant. Statistical analyses were conducted using IBM SPSS Statistics for Windows, version 26.0, (IBM Corp., Armonk NY, USA) and R software, version R×64 4.1.0 (https://www.r-project.org/).

## Results

### Baseline Characteristics

The number of patients with DCI were 85 (28%) and 27 (27%) in training and validation cohorts, and women comprised 179 (59%) and 68 (67%) patients in the two groups, respectively. The median age in both the cohorts was 57 years. In terms of other admission clinical features, there were more patients with mFS of 3–4 point in the validation cohort than training cohort (*P* < 0.05), and the aneurysm mean length size in the validation cohort was larger than the training cohort (*p* < 0.05). Among the patients with aSAH in the validation cohort, there is a larger proportion of patients who chose aneurysm clipping (*p* < 0.05). However, there were no significant differences in medical history, disease history, other clinical conditions, aneurysm location, aneurysm number, admission laboratory results, and admission CT value between the two cohorts (*P* > 0.05). Table 1 shows the detailed baseline characteristics of the datasets. We also analyzed the baseline characteristics of the DCI and non-DCI groups in the training cohort. Table 2 shows the detailed baseline data of the two groups in the training cohort.

**Table 1:** Patinets baseline characteristics in training and validation cohorts.

**Characteristics**	**Training cohort** **(*n* = 303)**	**Validation cohort** **(*n* = 101)**	***P*-value**
**Demographics**			
Age (years)	57 (51, 64)	57 (51, 63)	0.903
Gender (Female)	179 (59)	68 (67)	0.175
**Medical history**			
Hypertension	142 (47)	50 (50)	0.73
Diabetes	9 (3)	3 (3)	1.000
CHD	12 (4)	2 (2)	0.532
Smoking	54 (18)	17 (17)	0.94
Drinking	37 (12)	13 (13)	1.000
Anticoagulant	11 (4)	3 (3)	1.000
Disease history			0.886
ICH	3 (1)	0 (0)	
CI	6 (2)	1 (1)	
WFNS grade			0.163
I–II	227 (75)	68 (67)	
III	37 (12)	13 (13)	
IV	22 (7)	12 (12)	
V	17 (6)	8 (8)	
Hunt and Hess grade			0.327
I–II	211 (70)	59 (58)	
III	59 (19)	27 (27)	
IV	18 (6)	9 (9)	
V	15 (5)	6 (6)	
Modified Fisher scale			0.037
1–2	157 (52)	36 (35)	
3	76 (25)	37 (37)	
4	70 (23)	28 (28)	
Aneurysm location			0.694
ACA	273 (90)	93 (92)	
PCA	30 (10)	8 (8)	
Aneurysm number			0.428
Single	269 (89)	86 (85)	
Multiple (≥2)	34 (11)	15 (15)	
**Mean aneurysm size**			
Neck (mm)	3.2 (2.4, 3.75)	3.5 (2.5, 4.3)	0.068
Length (mm)	4.4 (3.15, 5.5)	4.9 (3.5, 6.7)	0.017
Aneurysm treatment			0.015
Clipping	154 (51)	66 (65)	
Coiling	149 (49)	35 (35)	
Decompressive craniectomy	16 (5)	11 (11)	0.084
**Admission laboratory results**			
Glucose (mmol/L)	6.96 (5.94, 8.17)	6.8 (5.61, 8.06)	0.396
D-dimer (mg/L)	1.17 (0.58, 2.5)	1.36 (0.82, 2.5)	0.288
WBC (10^∧^9/L)	11.23 (9.27, 13.99)	11.21 (8.98, 14.4)	0.646
Neutrophil (10^∧^9/L)	9.57 (7.56, 12.28)	9.9 (7.27, 12.8)	0.887
Lymphocyte (10^∧^9/L)	0.9 (0.68, 1.25)	0.92 (0.65, 1.27)	0.842
Monocytes (10^∧^9/L)	0.5 (0.34, 0.7)	0.5 (0.33, 0.7)	0.655
**Admission CT value (HU)**			
ClotCT	57 (52, 62.02)	58 (54, 63)	0.17
EdemaCT	26.82 (24.2, 28.98)	26.88 (25.07, 29)	0.36
DCI	85 (28)	27 (27)	0.898

*DCI, indicates delayed cerebral ischemia; ACA, aneurysm includes anterior cerebral artery, middle cerebral artery, internal cerebral artery, anterior communicating artery; posterior communicating artery; PCA, aneurysm includes posterior cerebral artery, basilar artery, anterior inferior cerebellar artery, posterior inferior cerebellar artery, vertebral artery; ACA, anterior circulation aneurysm; PCA, posterior circulation aneurysm; WBC, White blood cell; CT, computer tomography; HU, Hounsfield Unit; WFNS, World Federation of Neurosurgical Surgeons; ICH, Intracerebral hemorrhage; CI, cerebral infarction; CHD, Coronary heart disease*.

**Table 2:** Patients baseline characteristics in model training cohort.

**Characteristics**	**Total (*n* = 303)**	**Non-DCI (*n* = 218)**	**DCI (*n* = 85)**	***P*-value**
**Demographics**				
Age (years)	57 (51, 64)	57 (52, 65)	56 (49, 64)	0.414
Gender (Female)	179 (59)	126 (58)	53 (62)	0.552
**Medical history**				
Hypertension	142 (47)	103 (47)	39 (46)	0.932
Diabetes	9 (3)	6 (3)	3 (4)	0.714
CHD	12 (4)	5 (2)	7 (8)	0.042
Smoking	54 (18)	35 (16)	19 (22)	0.263
Drinking	37 (12)	26 (12)	11 (13)	0.962
Anticoagulant	11 (4)	6 (3)	5 (6)	0.19
Disease history				0.367
ICH	3 (1)	2 (1)	1 (1)	
CI	6 (2)	3 (1)	3 (4)	
WFNS grade				<0.001
I–II	227 (75)	185 (85)	42 (49)	
III	37 (12)	22 (10)	15 (18)	
IV	22 (7)	7 (3)	15 (18)	
V	17 (6)	4 (2)	13 (15)	
Hunt and Hess grade				<0.001
I–II	211 (70)	167 (77)	44 (52)	
III	59 (19)	42 (19)	17 (20)	
IV	18 (6)	5 (2)	13 (15)	
V	15 (5)	4 (2)	11 (13)	
Modified Fisher scale				<0.001
1–2	157 (52)	126 (57)	31 (36)	
3	76 (25)	56 (26)	20 (24)	
4	70 (23)	36 (17)	34 (40)	
Aneurysm location				0.695
ACA	273 (90)	195 (89)	78 (92)	
PCA	30 (10)	23 (11)	7 (8)	
Aneurysm number				1.000
Single	269 (89)	194 (89)	75 (88)	
Multiple (≥2)	34 (11)	24 (11)	10 (12)	
**Mean aneurysm size**				
Neck (mm)	3.2 (2.4, 3.75)	3.2 (2.5, 3.98)	3.2 (2.2, 3.7)	0.228
Length (mm)	4.4 (3.15, 5.5)	4.4 (3.26, 5.5)	4.2 (3, 6)	0.963
Aneurysm treatment				0.002
Clipping	154 (51)	98 (45)	56 (66)	
Coiling	149 (49)	120 (55)	29 (34)	
Decompressive craniectomy	16 (5)	3 (1)	13 (15)	<0.001
**Admission laboratory results**				
Glucose (mmol/L)	6.96 (5.94, 8.17)	6.94 (5.92, 8.14)	6.96 (6.1, 8.3)	0.454
D-dimer (mg/L)	1.17 (0.58, 2.5)	1.11 (0.55, 2.33)	1.51 (0.78, 3.82)	0.024
WBC (10^∧^9/L)	11.23 (9.27, 13.99)	10.5 (8.75, 12.65)	14.6 (11.7, 17.3)	<0.001
Neutrophil (10^∧^9/L)	9.57 (7.56, 12.28)	8.89 (7.27, 11.16)	12.1 (9.67, 14.7)	<0.001
Lymphocyte (10^∧^9/L)	0.9 (0.68, 1.25)	0.94 (0.68, 1.26)	0.83 (0.68, 1.09)	0.266
Monocytes (10^∧^9/L)	0.5 (0.34, 0.7)	0.44 (0.32, 0.66)	0.65 (0.44, 0.9)	<0.001
**Admission CT value (HU)**				
ClotCT	57.1 ± 7.05	55.23 ± 6.33	61.9 ± 6.54	<0.001
EdemaCT	26.82 (24.2, 28.98)	26.75 (24.25, 28.45)	27 (24.2, 30)	0.28

*ACA, aneurysm includes anterior cerebral artery, middle cerebral artery, internal cerebral artery, anterior communicating artery; posterior communicating artery; PCA, aneurysm includes posterior cerebral artery, basilar artery, anterior inferior cerebellar artery, posterior inferior cerebellar artery, vertebral artery; ACA, anterior circulation aneurysm; PCA, posterior circulation aneurysm; WBC, White blood cell; CT, computer tomography; HU, Hounsfield Unit; WFNS, World Federation of Neurosurgical Surgeons; ICH, Intracerebral hemorrhage; CI, cerebral infarction; CHD, Coronary heart disease*.

### Model Performance Evaluation and Comparison

**Figure 2:**
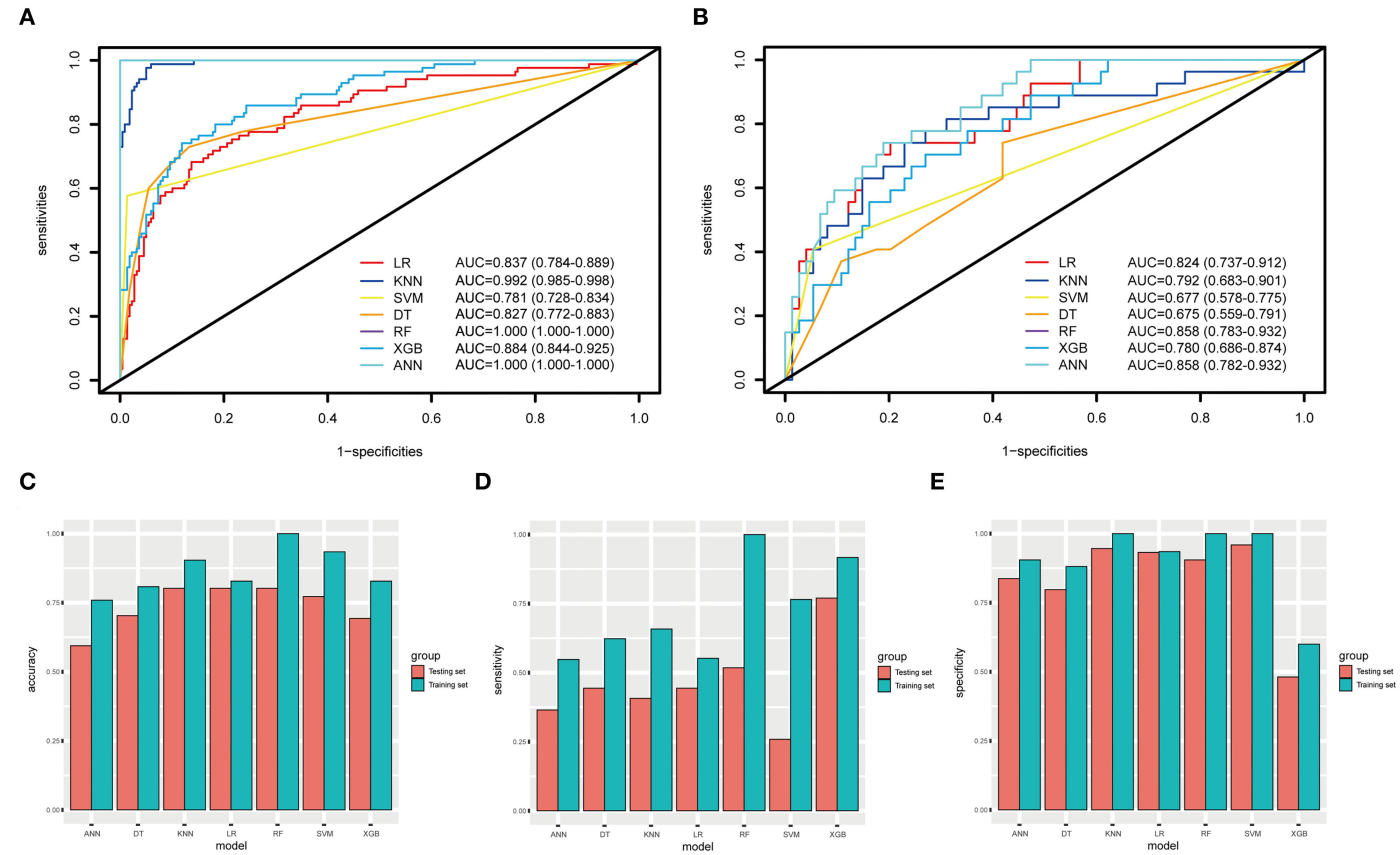
The ROC curves, accuracy, sensitivity, and specificity of ML methods and conventional LR. **(A,B)** The AUCs of ML methods and LR in the training and testing datasets. **(C–E)** The accuracy, sensitivity, and specificity of LR, KNN, SVM, DT, RF, XGB, and ANN in the training and testing datasets are 82.8, 90.4, 93.4%, 80.8, 100, 82.8, 75.9% (blue); 80.2, 80.2, 77.2, 70.3, 80.2, 69.3, 59.4% (orange); 55.2, 65.8, 76.5, 62.3, 100, 91.7, 54.8% (blue); 44.4, 40.7, 25.9, 44.4, 51.8, 77, 36.5% (orange), 93.5, 100, 100, 88.1, 100, 60, 90.5% (blue); 93.2,94.6, 95.9, 79.7, 90.5, 48.1, 83.7% (orange).

**Figure 3:**
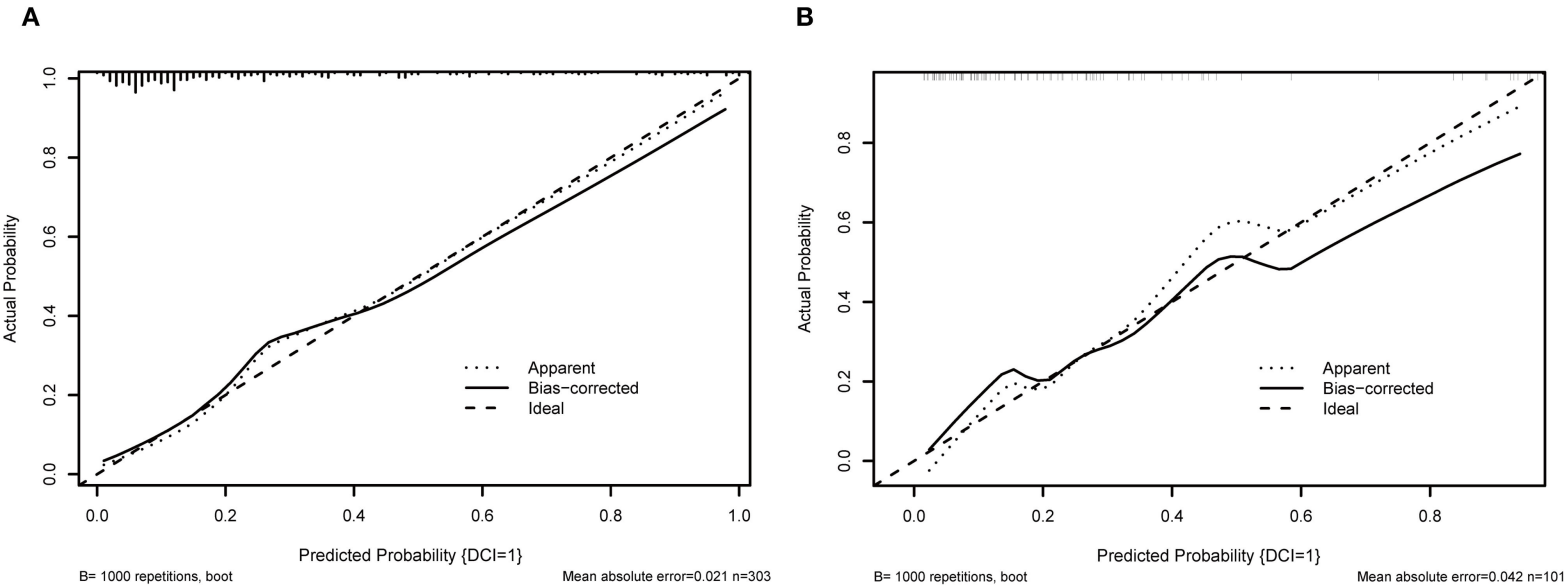
The calibration curve of RF model. **(A)** shows the 10-fold cross validation using training cohort; **(B)** illustrates the 10-fold cross-validation using the validation cohort.

**Figure 4:**
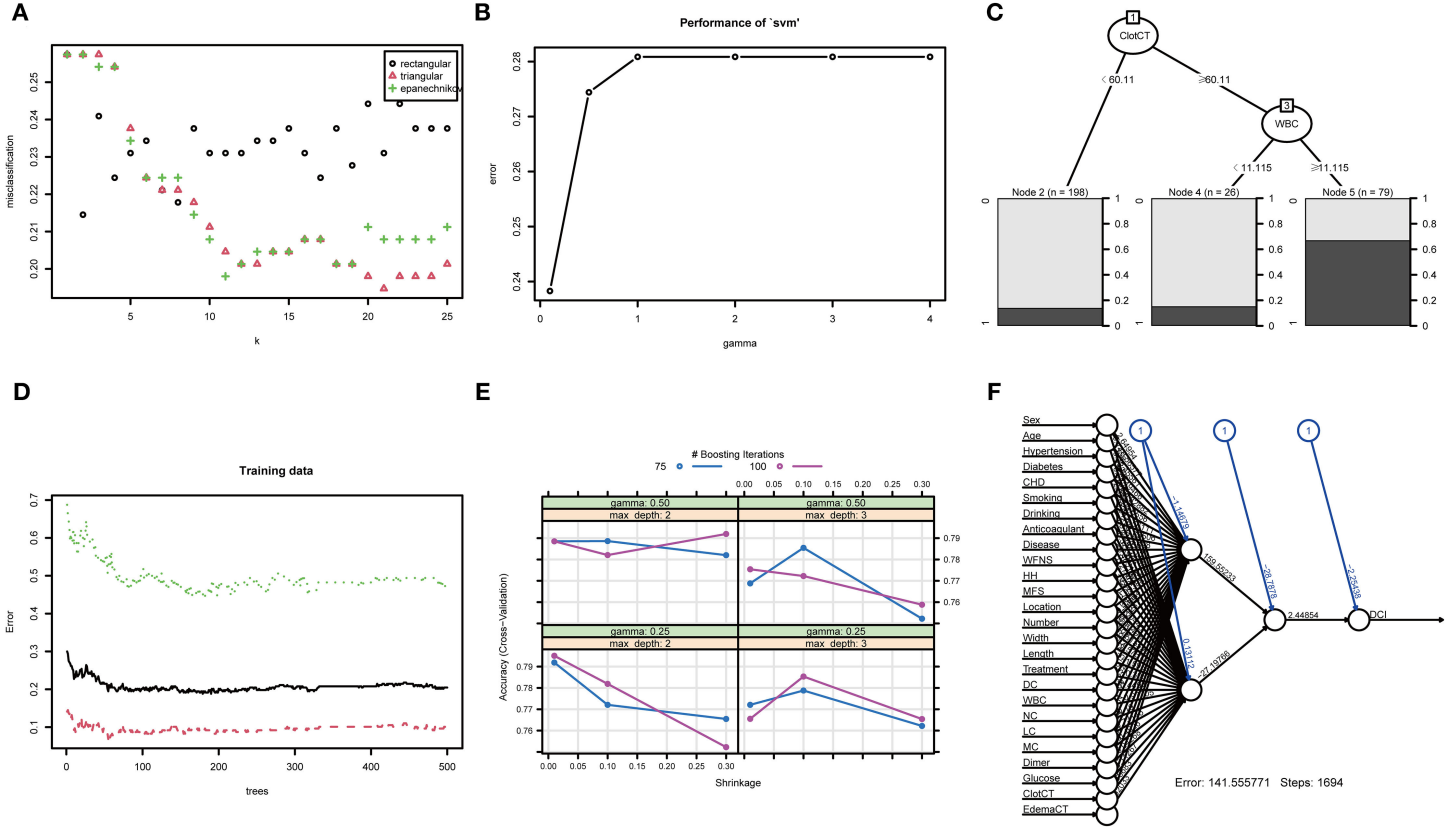
The training process and optimal parameters of six types of full-feature ML models. **(A)** shows the training process of k-nearest neighbor, and the optimal *K*-value is 21; **(B)** shows the error and gamma parameter of the support vector machine during the training process, and the two optimal parameters are 0.238 and 0.1; **(C)** illustrates the training process of the decision tree model, and the most important branches are subarachnoid clot CT value and WBC count; **(D)** displays the training process of random forest, and the optima tree number of the RF model is 179; **(E)** shows the training process of eXtreme Gradient Boosting, and the optimal parameters are gamma of 0.25, max depth of 2, and n-rounds value of 100; **(F)** demonstrates the training process of artificial neural network, and generalized weights of all clinical features are seen.

When using the validation cohort to evaluate and compare model performance, our results showed that conventional LR with an AUC value of 0.824 (95%CI: 0.73–0.91) outperformed KNN, decision tree, support vector machine, and extreme gradient boosting model with the AUCs of 0.792 (95%CI: 0.68–0.9, DeLong: *P* = 0.46), 0.675 (95%CI: 0.56–0.79, DeLong: *P* < 0.01), 0.677 (95%CI: 0.57–0.77, DeLong: *P* < 0.01), and 0.78 (95%CI: 0.68–0.87, DeLong: *P* = 0.50). However, the RF and ANN model with a same AUC (0.858, 95%CI: 0.78–0.93, DeLong: *P* = 0.26) still performed well than the LR. Furthermore, the accuracy and balanced accuracy of the RF were 20.8 and 11% higher than the latter. Supplementary Table 1 shows the confusion matrix and balanced accuracy of ML and LR model using training and validation cohorts. Figure 2 and Table 3 present the performances of all models when using the training and validation cohorts. In addition, Figure 3 demonstrates that the superior RF model had a good calibration performance according to the calibration curve and Hosmer–Lemeshow test in the training (X^2^ = 8.78, df = 8, *P*-value = 0.36;) and validation cohort (X^2^ = 10.97, df = 8, *P*-value = 0.203). Table 4 and Figure 4 show the process of model development.

**Table 3:** Model performance evaluation using training and validation cohorts.

**Cohort**	**Model**	**AUC (95%CI)**	**Accuracy**	**Sensitivity**	**Specificity**
Training	LR	0.837 (0.784–0.889)	0.828	0.552	0.935
	KNN	0.992 (0.985–0.998)	0.904	0.658	1.000
	SVM	0.781 (0.728–0.834)	0.934	0.765	1.000
	DT	0.827 (0.772–0.883)	0.808	0.623	0.881
	RF	1.000 (1.000–1.000)	1.000	1.000	1.000
	XGB	0.884 (0.844–0.925)	0.828	0.917	0.600
	ANN	1.000 (1.000–1.000)	0.759	0.548	0.905
Validation	LR	0.824 (0.737–0.912)	0.802	0.444	0.932
	KNN	0.792 (0.683–0.901)	0.802	0.407	0.946
	SVM	0.677 (0.578–0.775)	0.772	0.259	0.959
	DT	0.675 (0.559–0.791)	0.703	0.444	0.797
	RF	0.858 (0.783–0.932)	0.802	0.518	0.905
	XGB	0.780 (0.686–0.874)	0.693	0.77	0.481
	ANN	0.858 (0.782–0.932)	0.594	0.365	0.837

*LR, logistic regression; KNN, K-nearest neighbor; SVM, support vector machine; DT, decision tree model; RF, random forest; XGBoost, extreme gradient boosting; ANN, artificial neural network*.

**Table 4:** The univariate and multivariate analysis during fitting logistic regression model.

**Variable**	**OR (95%CI)**	***P*-value**	**Variable**	**aOR (95%CI)**	***P*-value**
CHD	3.82 (1.18–13.25)	0.025	*NA*	*NA*	*NA*
WFNS	2.07 (1.65–2.63)	<0.001	WFNS	1.53 (1.11–2.12)	0.009*
HH	1.97 (1.53–2.58)	<0.001	*NA*	*NA*	*NA*
MFS	1.57 (1.24–2.02)	<0.001	MFS	0.76 (0.53–1.08)	0.137
Treatment	0.42 (0.24–0.71)	0.001	Treatment	0.41 (0.21–0.77)	0.007*
DC	12.93 (4.03–57.6)	<0.001	DC	4.54 (1.01–25.13)	0.059
ClotCT	1.17 (1.12–1.23)	<0.001	ClotCT	1.11 (1.05–1.17)	<0.001*
WBC	1.29 (1.19–1.39)	<0.001	WBC	1.58 (1.11–2.33)	0.018*
NC	1.25 (1.16–1.36)	<0.001	NC	0.74 (0.5–1.07)	0.141
MC	8.44 (3.48–21.59)	<0.001	*NA*	*NA*	*NA*
D-dimer	1.07 (1.02–1.15)	0.016	*NA*	*NA*	*NA*

*CHD, Coronary heart disease; WFNS, World Federation of Neurosurgical Surgeons; HH, Hunt and Hess grade; MFS, modified Fisher scale; DC, Decompressive craniectomy; WBC, White blood cell; NC, Neutrophil; MC, Monocytes. aOR, adjusted odds ratio.*

* *indicates statistical significance (p < 0.05) by multivariate logistic regression*.

### Individual Variable Importance

**Figure 5:**
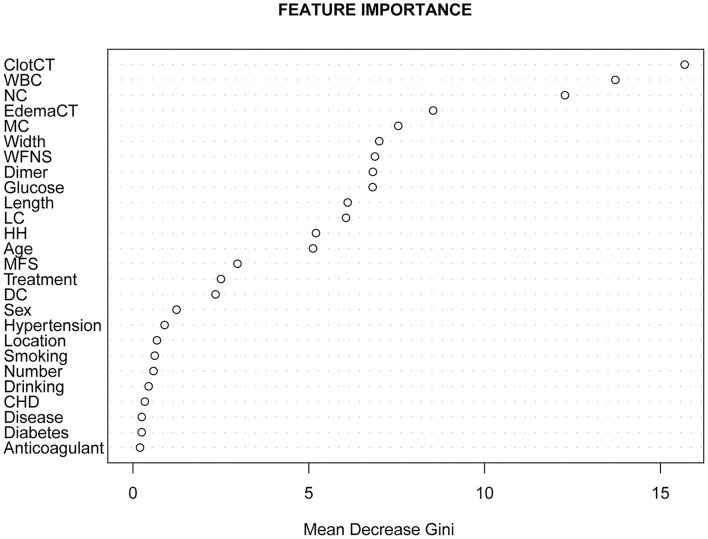
All features importance of random forest calculated by Gini index.

**Figure 6:**
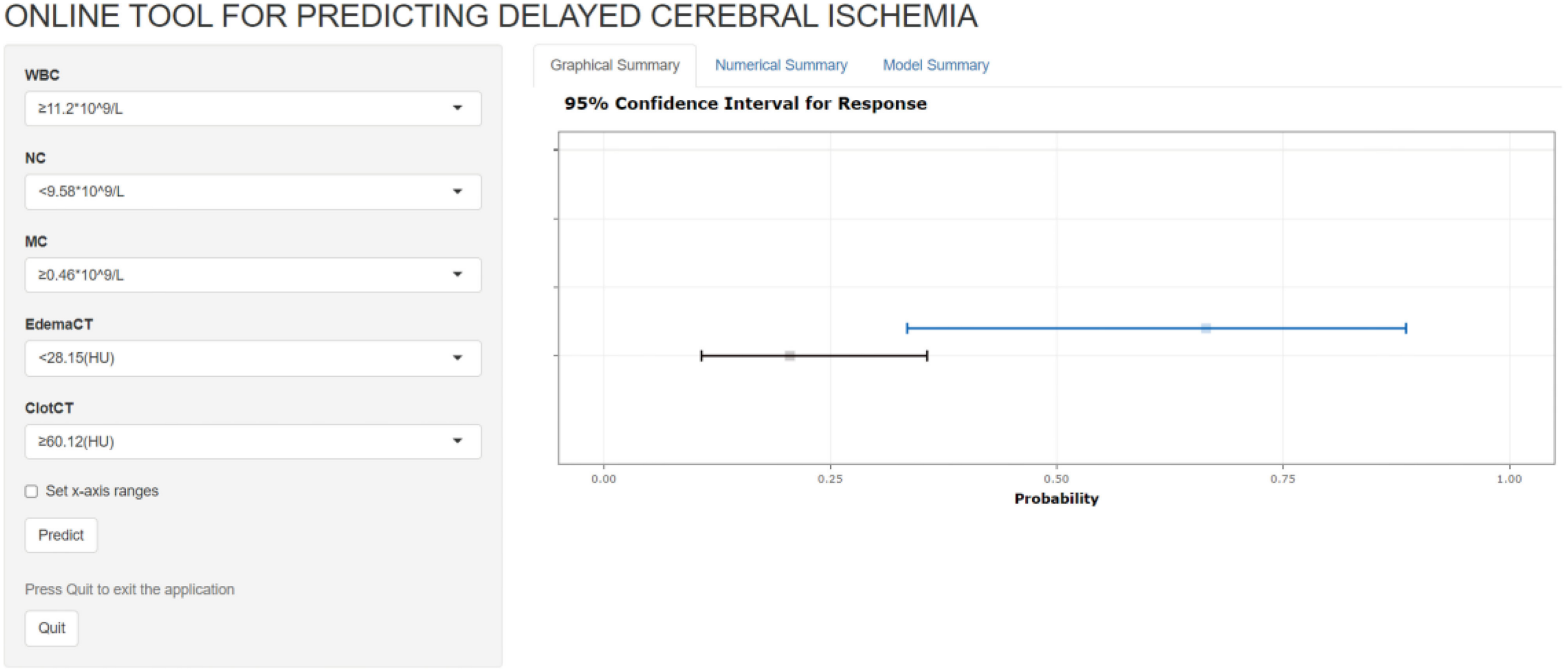
The interface of the online prediction tool for predicting delayed cerebral ischemia.

**Figure 7:**
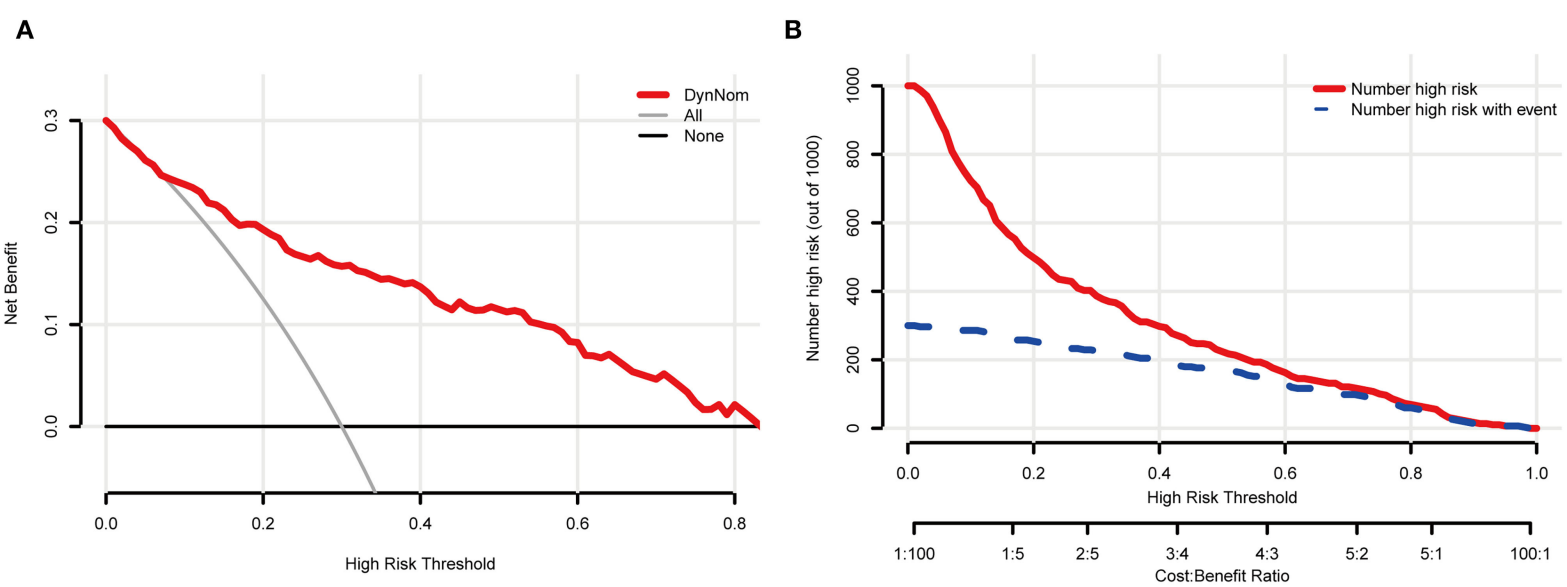
The clinical and practical evaluation of the online prediction tool. **(A)** shows a decision curve analysis (DCA); **(B)** displays a clinical impact curve (CIC).

The five most important features for DCI prediction were CT value of subarachnoid hemorrhage (15.68), WBC count (13.72), neutrophil count (12.28), CT value of cerebral edema (8.54), and monocyte count (7.54). The cut-off value of WBC, neutrophil, and monocyte counts for predicting DCI were 11.2 × 10^∧9^/L, 9.58 × 10^∧9^/L, and 0.46 × 10^∧9^/L, respectively. Moreover, the cut-off value of CT value in subarachnoid hemorrhage and cerebral edema were 60.12 (HU) and 28.15 (HU). Figure 5 shows all input feature importance. An online prediction tool (https://dynamic-nomogram.shinyapps.io/DynNomapp-DCI/) was developed based on the five optimal predictors in the RF model, which could precisely calculate the risk value of DCI after aSAH. A risk percentage of 50% calculated by this tool commonly represents an occurrence of DCI in patients with aSAH. Figure 6 displays the interface of the online tool for predicting DCI. Both decision curve analysis and clinical impact curve on the validation cohort showed a superior overall net benefit over the entire range of threshold probabilities (Figure 7).

## Discussion

In this study, the eligible patients with aSAH from five medical centers were randomly divided into model training and validation cohorts. One conventional LR and six types of famous ML methods were used to construct the prediction model by incorporating relatively complete admission clinical data, and all model performances were assessed and compared. To the best of our knowledge, this study is the first to utilize the rounded clinical features to develop the model and systematically compare the performance of several popular ML methods and conventional LR on DCI prediction. In addition, firstly, we developed an online prediction tool based on the most important features of the RF model to precisely calculate the risk of DCI development.

It was considered that only a few admission clinical features would not lead to an accurate DCI prediction. However, the most commonly used multivariable prediction models are still based on LR. For instance, de Rooij et al. (2013) incorporated some features selected by LR and constructed a practical risk chart for DCI prediction. The AUC value of this risk chart was 0.69 in the validation cohort. Liu et al. (2020) used six factors selected *via* LR to develop a nomogram for DCI, which achieved an AUC value of 0.65 on the test set. Other studies have also employed the conventional LR method to identify independent factors for DCI prediction (Al-Mufti et al., 2017, 2019a; Duan et al., 2018; Hurth et al., 2020). In our study, the LR model incorporated four independent features for DCI classification and achieved an AUC value of 0.837 in the validation cohort, which was higher than the AUC values previous models reported (de Oliveira Manoel et al., 2015; Foreman et al., 2017; van der Steen et al., 2019; Liu et al., 2020). The inclusion of complete admission clinical information can enable the LR to select the optimal variables to improve the prediction performance, which may explain the better performance of our LR model. However, owing to the robustness of the LR model, it cannot take full advantage of information from all clinical input features.

Machine learning models can solve the problem of high-dimensional data more robustly than the conventional LR method, making them suitable for fitting more features for prediction (Brusko et al., 2018; Buchlak et al., 2020). This capability can reduce the subjectivity in statistical analysis and ensure the objectivity of the results. Recently, ML algorithms have been developed rapidly, and some studies have reported the use of ML to predict the occurrence of DCI. de Jong et al. (2021) constructed a feedforward artificial neural network model and achieved an AUC of 0.72 for DCI prediction with a database with 362 patients. Their model performed equally well as the VASOGRADE model (de Oliveira Manoel et al., 2015). The ANN model in our study, with an AUC of 0.858, had a better predictive power than the conventional LR model and outperformed the previous ANN model.

Some researchers have compared the performances of LR and ML models for the prediction of DCI or other diseases. For instance, Savarraj et al. (2021) developed ML and LR models for DCI classification using a dataset with 399 patients. Their results showed that the ML model with the highest AUC value of 0.75 ± 0.07 outperformed the LR model. Ramos et al. (2019) reported that the ML model with the highest AUC value of 0.74 performed better than the best LR model with an AUC of 0.63. However, Nusinovici et al. (2020) reported that the LR model could perform equivalently to the ML models in their study, and Chen et al. (2020) showed that ML models cannot outperform the conventional LR model in predicting other diseases. In our study, we constructed several popular ML models based on the relatively complete clinical features, some of which were not compared in previous studies. The prediction ability of the LR model was inferior to those of the ANN and RF models, but better than those of the KNN, support vector machine, decision tree, and extreme gradient boosting models. This indicates that the traditional LR method still can play an important role in DCI prediction. Although ML can make perfect use of the input characteristics, data overfitting may lead to poor prediction performance.

Subarachnoid hemorrhage is a state of systemic inflammatory response syndrome, with both biochemical and cellular reactions (Parkinson and Stephensen, 1984). SAH initiates the rapid activation of the inflammatory cascade, and growing evidence suggests that an early neurovascular inflammatory response is a potential mechanism of late cerebral vasospasm and early brain injury (Helbok et al., 2015). The CT value in SAH often represents the subarachnoid clot density and can reflect the cerebral inflammatory response. At present, the measurement method of CT density value of subarachnoid clot still relies on the manual drawing of ROI. Kanazawa et al. (2020) found that an ROI CT value of ≥49.95 HU is correlated with DCI occurrence. Our results are consistent with those of previous studies showing that the CT value of >60.12 HU plays a prominent role in DCI prediction. Additionally, Ahn et al. (2018) constructed a scoring system for predicting DCI and clinical outcomes based on early cerebral edema after aSAH. This scoring system may become a surrogate marker of early brain injury and predicts DCI and prognosis after aSAH. Our consequence also illustrates that early cerebral edema also has an important influence on DCI prediction. As we know, WBC and neutrophil counts also play an important role in reflecting neuroinflammatory responses. Al-Mufti et al. (2019b) found that a WBC count >12.1 × 10^9^/L was the strongest predictor of DCI after adjusting for confounding factors, including clinical grade and aneurysm clipping treatment. Our results found that the WBC count >11.2 × 10^∧9^/L, neutrophil count >9.58 × 10^∧9^/L, and monocytes count >0.46 × 10^∧9^/L were the most important features for the prediction DCI. A recent study has shown that admission WBC, neutrophil, and monocyte counts were higher in patients with DCI and unfavorable prognosis (Gusdon et al., 2021). Inspiringly, our study confirmed this, which could account for the fact that DCI development is closely relative to the inflammatory response. Future basic research should further explore the inflammatory machine during the occurrence of DCI.

Based on the superior prediction performance of the RF, we used the most important features to construct an online prediction tool, which will aid in the early identification of patients at high risk of DCI after aSAH and allow timely interventions.

Our study systematically collected admission baseline information, laboratory test results, and admission CT imaging data, and these pieces of information are representative as possible of the true condition of aSAH patients when they are admitted to the hospital. Secondly, in order to avoid the defects of single-center data modeling, we collected data from multiple medical centers, making the DCI prediction model more generalized and robust, which is the second innovation of this study. Thirdly, this study covers several of the most popular machine learning algorithms, which have not been systematically compared with conventional models in previous studies, which is also an innovation point. Fourth, we built an online version of the prediction tool, which is convenient for clinicians to calculate the risk of DCI based on patient information at admission. However, there are several limitations that were observed. This was a retrospective study, and a larger prospective study should be considered to validate our results. Second, a possible deviation caused by manual ROI drawing is unavoidable. The agreement measurements for CT values between an experienced neurosurgeon and a radiologist were acceptable. Third, having an accuracy of 1 or AUC of 1 on the training dataset means the model is perfect, which is clearly not the case. Among the model we constructed, the random forest has overfitting. We know that overfitting may occur when the model tries to fit all the predicted features with a limited training dataset, which is to say a modeling error in statistics that occurs when a function is too closely aligned to the training dataset. Our future studies will collect more samples to further verify the results of the RF mode.

## Conclusions

In this multicenter study, we found that several ML methods, particularly random forest, outperformed conventional LR. Furthermore, an online prediction tool based on the random forest model was developed to identify patients at high risk for delayed cerebral ischemia after subarachnoid hemorrhage and facilitate timely interventions.

## Data Availability Statement

The raw data that supporting the findings of this study are available from the corresponding author upon reasonable request.

## Ethics Statement

The Medical Ethics Committee of Renmin Hospital of Wuhan University (our principal affiliation site) approved the study protocol (approval number WDRM2021-K022). The Ethics Committees of Huzhou Central Hospital (202108005-01), the Affiliated Hospital of Panzhihua University (202105002), General Hospital of Northern Theater Command (Y2021060), and the First Hospital of Shanxi Medical University (2021-Y6) also approved the protocol. The Medical Ethics Committee waived the need for patient consent because the data were derived from the electronic health record system. The patients/participants provided their written informed consent to participate in this study.

## Author Contributions

PH, YLi, HZ, and QC: study design. PH, YLi, LY, and YX: literature search. PH, YLi, YLiu, GG, XG, ZS, YS, and QS: data acquisition. PH, SY, YQ, and ML: data analysis and statistical analysis. PH, YLi, HZ, YQ, LW, GD, and QC: manuscript preparation, editing, and review. GD, ML, and XN: funding supporting. All authors read and approved the final manuscript.

## Funding

This work was supported by the National Natural Science Foundation of China (No. 82001311), the National Natural Science Foundation of China (No. 81971870), the National Natural Science Foundation of China (No. 81703752), and the National Natural Science Foundation of China (No. 82001385).

## Conflict of Interest

The authors declare that the research was conducted in the absence of any commercial or financial relationships that could be construed as a potential conflict of interest.

## Publisher's Note

All claims expressed in this article are solely those of the authors and do not necessarily represent those of their affiliated organizations, or those of the publisher, the editors and the reviewers. Any product that may be evaluated in this article, or claim that may be made by its manufacturer, is not guaranteed or endorsed by the publisher.
