# Supplementary material for: Comparison of Conventional Logistic Regression and Machine Learning Methods for Predicting Delayed Cerebral Ischemia After Aneurysmal Subarachnoid Hemorrhage: A Multicentric Observational Cohort Study
Source: Front Aging Neurosci. 2022 Jun 17;14:857521. doi: 10.3389/fnagi.2022.857521 (PMC9247265; doi:10.3389/fnagi.2022.857521)
Supplement: Supplementary file 1 [file Table_1.docx]

Supplementary Table 1. Confusion matrix of ML and LR models using training and validation cohorts

| Cohort* | Model | TP | FN | TN | FP | Ba-Acc |
| --- | --- | --- | --- | --- | --- | --- |
| Training | LR  KNN | 47  56 | 38  29 | 204  218 | 14  0 | 0.743  0.829 |
|  | SVM | 65 | 20 | 218 | 0 | 0.882 |
|  | DT | 53 | 32 | 192 | 26 | 0.752 |
|  | RF | 85 | 0 | 218 | 0 | 1 |
|  | XGB | 78 | 7 | 131 | 87 | 0.758 |
|  | ANN | 47 | 38 | 197 | 21 | 0.726 |
| Validation | LR  KNN | 12  11 | 15  16 | 69  70 | 5  4 | 0.688  0.676 |
|  | SVM | 7 | 20 | 71 | 3 | 0.609 |
|  | DT | 12 | 15 | 59 | 15 | 0.62 |
|  | RF | 14 | 13 | 67 | 7 | 0.711 |
|  | XGB | 21 | 6 | 36 | 38 | 0.625 |
|  | ANN | 10 | 17 | 62 | 12 | 0.601 |

LR indicates logistic regression; KNN, K-nearest neighbor; SVM, support vector machine; DT, decision tree model; RF, random forest; XGBoost, extreme gradient boosting; ANN, artificial neural network; TP, true positive; FN, false negative; TN, true negative; FP, false positive; Ba-Acc, balance accuracy
